# Small Cofactors May Assist Protein Emergence from RNA World: Clues from RNA-Protein Complexes

**DOI:** 10.1371/journal.pone.0022494

**Published:** 2011-07-18

**Authors:** Liang Shen, Hong-Fang Ji

**Affiliations:** Shandong Provincial Research Center for Bioinformatic Engineering and Technique, Shandong University of Technology, Zibo, People's Republic of China; University of Nottingham, United Kingdom

## Abstract

It is now widely accepted that at an early stage in the evolution of life an RNA world arose, in which RNAs both served as the genetic material and catalyzed diverse biochemical reactions. Then, proteins have gradually replaced RNAs because of their superior catalytic properties in catalysis over time. Therefore, it is important to investigate how primitive functional proteins emerged from RNA world, which can shed light on the evolutionary pathway of life from RNA world to the modern world. In this work, we proposed that the emergence of most primitive functional proteins are assisted by the early primitive nucleotide cofactors, while only a minority are induced directly by RNAs based on the analysis of RNA-protein complexes. Furthermore, the present findings have significant implication for exploring the composition of primitive RNA, *i.e.*, adenine base as principal building blocks.

## Introduction

The origin of life is undoubtedly a fundamental problem of natural science and attracts extensive attention of specialists in different fields of science. As RNA is the only known macromolecule acting as a genetic material as well as a catalyst, it is popular to think of early evolution of life progressed from an “RNA World”, in which RNA played a central role before protein and DNA emerged [1]–[5]. The “RNA world” hypothesis [3] has been widely discussed and at present there are no serious alternatives to an RNA world being one essential intermediate stage in the origin of life [6]–[8]. These studies have enabled us to resolve many pieces of the puzzle but have also left some critical gaps. For instance, a consequence of the RNA world model is that proteins have gradually replaced RNAs in catalysis by virtue of their superior catalytic properties over time [1], [7]. However, how primitive functional proteins emerged from the RNA world remains obscure.

Considering the fact that RNA may not serve simply as a molecular scaffold for protein folding but also may influence the function of a protein [8], it is rational to expect that in the RNA world the primitive functional protein emergence was directly induced by RNA molecules. If this conjecture was true, the structural information of primitive functional proteins replacing RNA molecules can be conceived in the present RNA-protein complexes. In view of the high conservation of the protein folds, the systematic analysis on the corresponding folds of RNA-binding proteins may help characterize the potential primitive proteins interacting with RNAs. In the present study, we proposed that the early primitive nucleotide cofactors play a major role in the primitive functional protein emergence. The present findings also have significant implication for understanding the composition of primitive RNA.

## Materials and Methods

The NPIDB, *i.e.*, Nucleic Acids–Protein Interaction DataBase [9], is a well-defined database that contains structures of RNA–protein complexes extracted from PDB, in which RNA–protein complexes were defined and selected according to the following criteria. First of all, the chains of RNA and protein in the coordinate (ATOM and HETATM) section of a PDB file are identified directly and only if a PDB file contains at least one protein chain and one RNA chain, it is included in NPIDB. Moreover, the interactions between RNAs and proteins in complexes are carefully examined. The hydrophobic interactions are evaluated by the CluD program [10], [11] and the recommended threshold distance range of hydrophobic interaction between a RNA and a protein is 4.5∼5.4 Å. The potential hydrogen bonds between a RNA and a protein are also detected based on the criterion that the distance between oxygen or nitrogen atoms of different molecules is <3.7 Å [9]. Taken together, the clear definition for the RNA-protein complexes in NPIDB guarantees the repeatability of the present analysis.

Until August 15 2010, 832 structures of RNA–protein complexes are available in NPIDB [9]. As our interest focused on the RNA-binding proteins with commented fold information, 184 structures were eliminated. Then, the corresponding folds of 648 RNA-binding proteins were identified manually from Structural Classification of Proteins (SCOP) database 1.75 [12], [13], which were classified into 177 types of domains and 134 families. Domain is defined as a part of protein sequence and structure, which can form a compact three-dimensional structure and can evolve, function, and exist independently of the rest of the protein chain [14]. For a little part of RNAs that are shared by two domains, both domains were counted. According to SCOP 1.75 [12], [13], these domains belong to 91 folds.

## Results

### Architectural profiles of RNA-binding proteins

**Figure 1:**
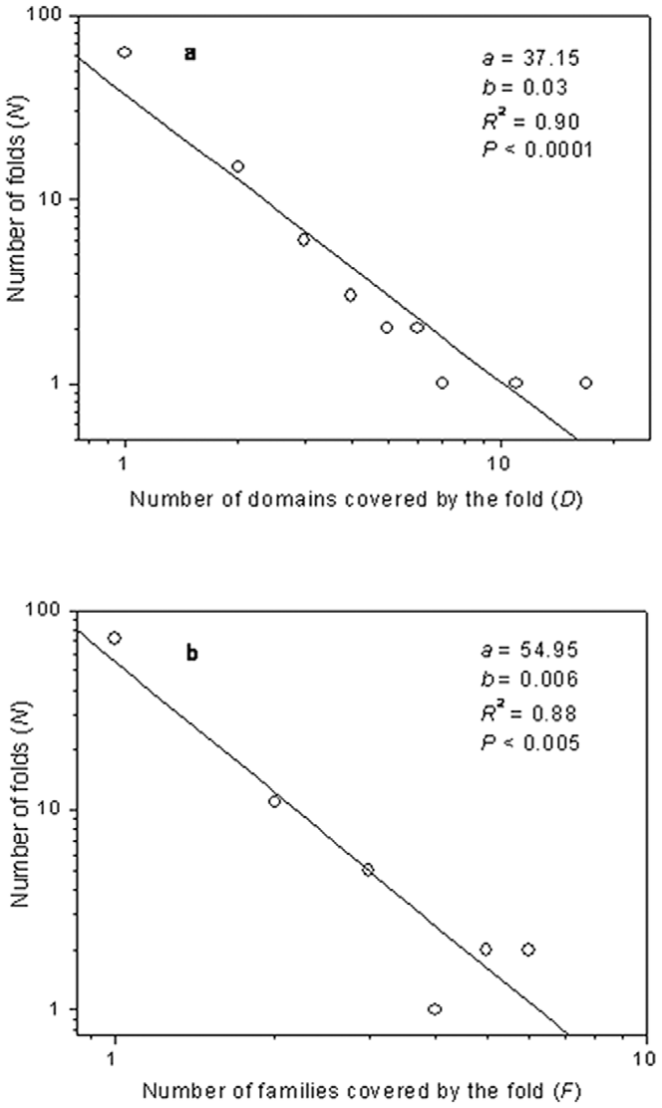
Power-law behaviors of RNA-protein binding. (**a**) The number of folds (*N*) decays with the increase of the number of RNA-binding domains covered by the fold (*D*) and follows power-law equation *N* = *aD^−b^* (*P*<0.0001). (**b**) The number of folds (*N*) decays with the increase of the number of RNA-binding families covered by the fold (*F*) and follows power-law equation *N* = *aF^−b^* (*P*<0.005).

As shown in Table S1, 91 folds do not distribute evenly in domain space. For instance, 60 folds (65.9%) appear only once in domain space, while 2 folds (2.2%) cover more than 10 domains respectively. Among 91 folds, Ferredoxin-like (d.58, which belongs to alpha and beta (a+b) protein class) is the most common fold, which cover 17 domains. Moreover, as illustrated in Fig. 1a, the number of folds (*N*) decays with the increase of the number of domains covered by the fold (*D*) and follows power-law equation *N* = *aD^−b^* (*P*<0.0001). As families are more evolutionarily conserved than domains, we also analyzed the distribution of the folds in family space and it was found that the correlation between the number of folds (*N*) and the number of families (*F*) also follows the power-law behavior (Fig. 1b).

### Biological basis underlying the power-law behaviors of RNA-binding protein architectures

The power-law behaviors of protein folds and ligand-protein binding have been successfully explained in terms of preferential attachment principle [15]–[18], which implicates that the more popular the folds, the earlier they originated. Therefore, it is desirable to explore the applicability of preferential attachment principle for the evolution of RNA-binding protein architectures. The age assignment for RNA-binding protein architectures are as follows: early RNA-binding protein architectures defined as those owned by both prokaryotic (*E. coli*) and eukaryotic (yeast or higher) species, while late architectures defined as those owned only by eukaryotic (yeast or higher) species. During the age-assigning process, to guarantee that the conclusion is not confined by the limited three-dimensional structures recorded in PDB, not only the proteins recorded in PDB were checked, but also the corresponding homologous proteins retrieved from Swiss-Prot database [19] were considered.

As shown in Table S1, 40 folds are owned by both prokaryotic (*E. coli*) and eukaryotic (yeast or higher) species, which suggests that these folds appeared relatively early. In comparison, 19 folds are only owned by eukaryotic (yeast or higher) species, which implies that these folds originated relatively late. Further analysis showed that early folds cover 2.8 domains on average, in contrast to the late folds that only cover 1.4 domains averagely. These results suggest that preferential attachment principle is indeed applicable to elucidating the power-law behaviors of RNA-binding protein architectures.

## Discussion

### Identification of the most ancient RNA-binding protein architecture

Based on the power-law feature of RNA-binding protein architectures and the underlying preferential attachment principle implication that the more widely shared architectures have an earlier origin, we can conclude that the higher occurrence of Ferredoxin-like (d.58) than others in RNA-binding domain space implies that the most ancient RNA-binding proteins were very likely to adopt this fold. This opinion is partially supported by previous studies [20], [21]. Through analyzing the sequences, Hall *et al.* and Wächtershäuser proposed that ferredoxins should play an important role in the origin of life and may have been among the earliest proteins formed. Further inspection to the structural information of ferredoxins reveals that they have only iron and inorganic sulphur in their active sites, which is unlike to other electron-transfering proteins, *e.g.*, cytochromes and flavoproteins, employing complex organic molecules as cofactors. Thus, considering the abundance of iron and sulphur in the primitive Earth, ferredoxin should possess inherent advantage than others to emerge readily.

### Implications for tracing the emergence of primitive functional proteins

Through a large-scale phylogenomic analysis on 174 proteomes, Caetano-Anollés and co-workers established a chronology for proteins, in which 776 folds are recorded according to their evolutionary order, respectively. (http://www.manet.uiuc.edu/download/foldAncestryVal2_0.txt) [22]–[24]. According to the evolutionary sequence of 776 folds, Ferredoxin-like (d.58) is No. 5. It is interesting to note that this fold is indeed one of the earliest protein architectures. However, there are still four kinds of folds appeared earlier than d.58. How did the proteins of these four folds appear?

To address this question, the first ten most ancient folds characters were investigated. According to the MANET database [24], the first ten most ancient folds are as follows: P-loop containing nucleoside triphosphate hydrolases (c.37), DNA/RNA-binding 3-helical bundle (a.4), TIM beta/alpha-barrel (c.1), NAD(P)-binding Rossmann-fold domains (c.2), Ferredoxin-like (d.58), Flavodoxin-like (c.23), Ribonuclease H-like motif (c.55), OB-fold (b.40), S-adenosyl-L-methionine-dependent methyltransferases (c.66) and Adenine nucleotide alpha hydrolase-like (c.26). Interestingly, through examining the structures and functions of proteins belonging to these ten folds, we found that 8 of 10 folds are dominated by special cofactors. For instance, in c.37, ATP (adenosine-5′-triphosphate) is the most popular cofactor, which covers 17 of 24 families in whole. In turn, NAD (nicotinamide-adenine-dinucleotide)/FAD (flavin-adenine dinucleotide)/FMN (flavin mononucleotide) for c.1, NAD(P) for c.2, FMN for c.23, AT(D)P for c.55, THP (thymidine-3′,5′-diphosphate) for b.40, SAH (S-adenosyl-L-homocysteine) for c.66 and AMP (adenosine monophosphate) for c.26. Considering the prevalent cofactor-induced protein folding [25]–[29], we hypothesize that the early cofactors may facilitate primitive functional protein formation. If this hypothesis is reasonable, some cofactors should be, at least in some cases, still be covalently linked to the protein component. Indeed, there are many such cases in which the cofactors contained both nucleotide and amino acid characteristics (Table 1) [30]–[32], which might be considered as the vestige of the ancient link between the cofactor and amino acid or protein. Furthermore, Szathmary *et al.* also suggested that amino acids were used by ribozymes (also called catalytic RNA) as cofactors in anticodon-plus-amino-acid complexes, in which, cofactors consisted of an amino acid bonded to one or more nucleotide (oligonucleotide) [33]–[36]. A close inspection on the structures of some cofactors, such as coenzyme A (CoA), FAD, NAD, and coenzyme F420, indicates that these should be sited at 5′ of RNAs, which provides further evidence to support the notion that early cofactors are the vestiges of RNA world [37]. Although the possibility cannot be excluded that random peptides existed in RNA world or preceded the RNA world, these random peptides should be much shorter than any used in life today and usually have no particular function. Therefore, according to the present results, we proposed that the origin of primitive functional proteins are mainly assisted by early primitive nucleotide cofactors. Despite RNAs may also participated in the emergence of primitive functional proteins, such as those in d.58, this pathway only plays a minor role in whole according to the analysis.

**Table 1:** Several cofactors contain both nucleotide and amino acid characteristics^a^.

Cofactors	Full name	Description^a^
CoA	Coenzyme A	ADP-pantothenylcysteamine
FAD	Flavin-adenine dinucleotide	Linked to lysine of proteins
FMN	Riboflavin-5′-phosphate	Linked to cysteine of proteins
SAM	S-adenosyl methionine	S-Adenosylmethionine
Factor 420	8-hydroxy-5-deazaflavin	Flavinoid-linked (Glu)2
F390-A	Adenosine 5′-phosphate	Flavinoid-linked (Glu)2
F390-G	Guanosine 5′- phosphate	Flavinoid-linked (Glu)2

a Most of the observations were taken from [30]–[32].

The present finding is also helpful for understanding the homochirality conundrum [38]–[42], *i.e.*, L-amino acids and D-sugars usually being preferred in nature over their respective enantiomers (mirror images) with the precondition of equal production of L- and D-amino acids provided at reaction equilibrium *in vitro*. Although L- and D-amino acids are proved to possess similar thermodynamic stability [43] and be equally efficient in building proteins [44], the preferential stabilization of the naturally occurring D-configuration of RNA over the L-configuration [45]–[48] is an inducement for proteins' selecting L-amino acid as their building blocks when early cofactors or RNA itself assisted the origin of primitive proteins.

### Implications for tracing the composition of the primitive RNA

An RNA world has been widely discussed as a probable stage in the early evolution of life [1]–[6], however, there are still several unanswered questions which highlight a dangerous weakness in the whole RNA world hypothesis. For instance, the pre-condition of RNA world hypothesis is that RNA molecules emerged in abiotic conditions, which implies that the building blocks of RNA, *i.e.*, adenine (A), cytosine (C), guanine (G), and uracil (U) were readily available on early prebiotic Earth. The isolation of adenine and guanine from meteorites can be act as evidence that these substances might have been available as “raw material” on early Earth [49], however, cytosine has neither been reported in such analyses nor is the product of electric spark discharge experiments [50]. Thus, it seems difficult for the primitive RNA to be constituted by four types of bases, *i.e.*, A, C, G, U.

Ribosomes, a place for protein synthesis, are large macromolecular assemblies consisting of RNAs and proteins, in which RNA plays a catalytic role in the formation of the peptide bond and the key catalytic site is proved to be only an adenine residue in the RNA [51], [52]. Further inspect showed that cofactors dominated in earliest folds, *e.g.*, ATP, NAD, FAD, ADP, SAH, AMP, contain a same base part, *i.e.*, adenine. Considering the notion that early cofactors are vestiges of RNA world, we thus believed that adenine base should be included in the original composition of primitive RNA. Moreover, many modern RNA molecules still contain adenine-rich sequences. For example, the adenine composition in a *Sendai virus* 18S messenger RNA is as high as 99.1% [53], which implies that simpler RNA still can perform its function. Based on the above results, we proposed that primitive RNA is most likely composed mainly by adenine base (A).

In summary, as a probable stage in the early evolution of life, RNA world has been wildely accepted because of the duplicate roles of RNA as both genetic material and catalysts. Thus, it is significant importance of tracing the pathway of RNA world to modern world. Although the possibility of random peptides existing in RNA world cannot be excluded, it was suggested that these random peptides usually have no particular function. Through systemic analysis of RNA-protein complexes, we proposed that primitive functional protein emergence is mainly assisted by early primitive nucleotide cofactors, while only a minority induced by RNA itself. The present findings also have significant implications for understanding origin of the homochirality of biomolecules and the composition of primitive RNA.

## Supporting Information

Table S1 **Folds, domains (number) and ownership of RNA-binding proteins.**
(DOC)
