# Supplementary material for: Small Cofactors May Assist Protein Emergence from RNA World: Clues from RNA-Protein Complexes
Source: PLoS One. 2011 Jul 18;6(7):e22494. doi: 10.1371/journal.pone.0022494 (PMC3138788; doi:10.1371/journal.pone.0022494)
Supplement: Table S1 — Folds, domains (number) and ownership of RNA-binding proteins. (DOC) [file pone.0022494.s001.doc]

**Supporting Information**

**Table S1**. Folds, domains (number) and ownership of RNA-binding proteins.

| **Fold** | **Ownership** | | **Family** | **Domain** |
| --- | --- | --- | --- | --- |
| **Ferredoxin-like (d.58)** | *E. coli* | *Homo sapiens* | 7 | **17** |
| **OB-fold (b.40)** | *E. coli* | *Homo sapiens* | 3 | **11** |
| **DNA/RNA polymerases (e.8)** | *E. coli* | *Homo sapiens* | 5 | **7** |
| **SH3-like barrel (b.34)** | *E. coli* | *Homo sapiens* | 3 | **6** |
| **Arg-rich RNA binding peptides (j.9)** | *-* | *Homo sapiens* | 6 | **6** |
| **DNA/RNA-binding 3-helical bundle (a.4)** | *E. coli* | *Homo sapiens* | 5 | **5** |
| **Nucleoplasmin-like/VP (viral coat and capsid proteins) (b.121)** | *E. coli* | *Homo sapiens* | 4 | **5** |
| **Four-helical up-and-down bundle (a.24)** | *E. coli* | *Mouse* | 2 | **4** |
| **Adenine nucleotide alpha hydrolase-like (c.26)** | *E. coli* | *Homo sapiens* | 1 | **4** |
| **Ribonuclease H-like motif (c.55)** | *E. coli* | *Homo sapiens* | 3 | **4** |
| **Anticodon-binding domain of a subclass of class I aminoacyl-tRNA synthetases (a.27)** | *E. coli* | *Yeast* | 1 | **3** |
| **Ribosomal protein L25-like (b.53)** | *E. coli* | *Homo sapiens* | 2 | **3** |
| **dsRBD-like (d.50)** | *E. coli* | *Yeast* | 1 | **3** |
| **Eukaryotic type KH-domain (KH-domain type I) (d.51)** | *E. coli* | *Homo sapiens* | 1 | **3** |
| **Rubredoxin-like (g.41)** | *E. coli* | *-* | 3 | **3** |
| **Ribosome and ribosomal fragments (i.1)** | *E. coli* | *Yeast* | 3 | **3** |
| **alpha-alpha superhelix (a.118)** | *-* | *Homo sapiens* | 2 | **2** |
| **Sm-like fold (b.38)** | *-* | *Homo sapiens* | 2 | **2** |
| **Reductase/isomerase/elongation factor common domain (b.43)** | *E. coli* | *Mouse* | 2 | **2** |
| **Ribosomal proteins L15p and L18e (c.12)** | *E. coli* | *Homo sapiens* | 1 | **2** |
| **Anticodon-binding domain-like (c.51)** | *E. coli* | *Homo sapiens* | 1 | **2** |
| **Microbial ribonucleases (d.1)** | *Aspergillus restrictus* | *-* | 2 | **2** |
| **Ribosomal proteins S24e, L23 and L15e (d.12)** | *E. coli* | *Homo sapiens* | 2 | **2** |
| **Ribosomal protein S5 domain 2-like (d.14)** | *E. coli* | *Homo sapiens* | 1 | **2** |
| **Alpha-L RNA-binding motif (d.66)** | *E. coli* | *Homo sapiens* | 2 | **2** |
| **Bacillus chorismate mutase-like (d.79)** | *-* | *Homo sapiens* | 1 | **2** |
| **Glucocorticoid receptor-like (DNA-binding domain) (g.39)** | *E. coli* | *Homo sapiens* | 2 | **2** |
| **Retrovirus zinc finger-like domains (g.40)** | *-* | *Homo sapiens* | 1 | **2** |
| **beta and beta-prime subunits of DNA dependent RNA-polymerase (e.29)** | *-* | *Homo sapiens* | 1 | **2** |
| **RNase III domain-like (a.149)** | *E. coli* | *Homo sapiens* | 2 | **2** |
| **Pseudouridine synthase (d.265)** | *E. coli* | *Homo sapiens* | 2 | **2** |
| **Long alpha-hairpin (a.2)** | *E. coli* | *-* | 1 | **1** |
| **Spectrin repeat-like (a.7)** | *E. coli* | *Homo sapiens* | 1 | **1** |
| **S15/NS1 RNA-binding domain (a.16)** | *E. coli* | *Homo sapiens* | 1 | **1** |
| **ROP-like (a.30)** | *Flock house virus* | *-* | 1 | **1** |
| **Signal peptide-binding domain (a.36)** | *E. coli* | *-* | 1 | **1** |
| **Ribosomal protein S7 (a.75)** | *E. coli* | *Homo sapiens* | 1 | **1** |
| **Ribosomal protein L19 (L19e) (a.94)** | *Haloarcula marismortui* | *-* | 1 | **1** |
| **An anticodon-binding domain of class I aminoacyl-tRNA synthetases (a.97)** | *-* | *Homo sapiens* | 1 | **1** |
| **Non-globular all-alpha subunits of globular proteins (a.137)** | *Haloarcula marismortui* | *-* | 1 | **1** |
| **Common fold of diphtheria toxin/transcription factors/cytochrome (b.2)** | *-* | *Homo sapiens* | 1 | **1** |
| **EV matrix protein (b.31)** | *Ebola virus* | *-* | 1 | **1** |
| **GroES-like (b.35)** | *Bacillus subtilis* | *-* | 1 | **1** |
| **Ribosomal protein L14 (b.39)** | *E. coli* | *Homo sapiens* | 1 | **1** |
| **Elongation factor/aminomethyltransferase common domain (b.44)** | *E. coli* | *Cow* | 1 | **1** |
| **Double-stranded beta-helix (b.82)** | *Bacillus subtilis* | *-* | 1 | **1** |
| **Barrel-sandwich hybrid (b.84)** | *E. coli* | *Homo sapiens* | 1 | **1** |
| **TIM beta/alpha-barrel (c.1)** | *Zymomonas mobilis* | *-* | 1 | **1** |
| **Barstar-like (c.9)** | *Haloarcula marismortui* | *-* | 1 | **1** |
| **Ribosomal protein L13 (c.21)** | *E. coli* | *Homo sapiens* | 1 | **1** |
| **Ribosomal protein L4 (c.22)** | *E. coli* | *Homo sapiens* | 1 | **1** |
| **Flavodoxin-like (c.23)** | *E. coli* | *Homo sapiens* | 1 | **1** |
| **Restriction endonuclease-like (c.52)** | *-* | *Homo sapiens* | 1 | **1** |
| **Formyltransferase (c.65)** | *E. coli* | *-* | 1 | **1** |
| **S-adenosyl-L-methionine-dependent methyltransferases (c.66)** | *Vaccinia virus* | *-* | 1 | **1** |
| **Periplasmic binding protein-like II (c.94)** | *E. coli* | *-* | 1 | **1** |
| **Cytidine deaminase-like (c.97)** | *E. coli* | *-* | 1 | **1** |
| **ssDNA-binding transcriptional regulator domain (d.18)** | *Trypanosoma brucei* | *-* | 1 | **1** |
| **Ribosomal protein S16 (d.27)** | *E. coli* | *Homo sapiens* | 1 | **1** |
| **Ribosomal protein S19 (d.28)** | *E. coli* | *Homo sapiens* | 1 | **1** |
| **Ribosomal protein L31e (d.29)** | *-* | *Homo sapiens* | 1 | **1** |
| **alpha/beta-Hammerhead (d.41)** | *-* | *Homo sapiens* | 1 | **1** |
| **Signal recognition particle alu RNA binding heterodimer, SRP9/14 (d.49)** | *-* | *Homo sapiens* | 1 | **1** |
| **Ribosomal protein S3 C-terminal domain (d.53)** | *E. coli* | *-* | 1 | **1** |
| **Ribosomal protein L22 (d.55)** | *E. coli* | *Mouse* | 1 | **1** |
| **Ribosomal protein L30p/L7e (d.59)** | *E. coli* | *-* | 1 | **1** |
| **RRF/tRNA synthetase additional domain-like (d.67)** | *E. coli* | *Mouse* | 1 | **1** |
| **RL5-like (d.77)** | *E. coli* | *Homo sapiens* | 1 | **1** |
| **RNA bacteriophage capsid protein (d.85)** | *Bacteriophage MS2* | *-* | 1 | **1** |
| **Class II aaRS and biotin synthetases (d.104)** | *-* | *Homo sapiens* | 1 | **1** |
| **Ribosomal protein S8 (d.140)** | *E. coli* | *Homo sapiens* | 1 | **1** |
| **Ribosomal protein L6 (d.141)** | *E. coli* | *Homo sapiens* | 1 | **1** |
| **Metallo-hydrolase/oxidoreductase (d.157)** | *E. coli* | *-* | 1 | **1** |
| **Ribosome inactivating proteins (RIP) (d.165)** | *-* | *Castor bean* | 1 | **1** |
| **Ribosomal protein L1 (e.24)** | *-* | *Homo sapiens* | 1 | **1** |
| **Beta-beta-alpha zinc fingers (g.37)** | *-* | *Homo sapiens* | 1 | **1** |
| **Reovirus components (i.7)** | *Reovirus* | *-* | 1 | **1** |
| **RNA polymerase (i.8)** | *-* | *Yeast* | 1 | **1** |
| **Computational models partly based on experimental data (i.11)** | *Thermotoga maritima* | *-* | 1 | **1** |
| **Ribosomal protein L10 (j.84)** | *-* | *Homo sapiens* | 1 | **1** |
| **NSP3 homodimer (e.34)** | *Simian 11 rotavirus* | *-* | 1 | **1** |
| **Small protein B (SmpB) (b.111)** | *Thermus thermophilus* | *-* | 1 | **1** |
| **S13-like H2TH domain (a.156)** | *E. coli* | *Homo sapiens* | 1 | **1** |
| **Nucleotidyltransferase (d.218)** | *Aquifex aeolicus* | *-* | 1 | **1** |
| **PUA domain-like (b.122)** | *Pyrococcus horikoshii* | *-* | 1 | **1** |
| **GatB/YqeY motif (a.182)** | *E. coli* | *-* | 1 | **1** |
| **CCCH zinc finger (g.66)** | *-* | *Homo sapiens* | 1 | **1** |
| **Tombusvirus P19 core protein, VP19 (d.255)** | *Tomato bushy stunt virus* | *-* | 1 | **1** |
| **Signal recognition particle (SRP) complex (i.22)** | *interspecies* | *-* | 1 | **1** |
| **Hut operon positive regulatory protein HutP (d.275)** | *Bacillus subtilis* | *-* | 1 | **1** |
| **Rhabdovirus nucleoprotein-like (a.260)** | ***Rabies virus*** | ***-*** | **1** | **1** |
